# Methods for Evaluating the Content, Usability, and Efficacy of Commercial Mobile Health Apps

**DOI:** 10.2196/mhealth.8758

**Published:** 2017-12-18

**Authors:** Danielle E Jake-Schoffman, Valerie J Silfee, Molly E Waring, Edwin D Boudreaux, Rajani S Sadasivam, Sean P Mullen, Jennifer L Carey, Rashelle B Hayes, Eric Y Ding, Gary G Bennett, Sherry L Pagoto

**Affiliations:** ^1^ Division of Preventive and Behavioral Medicine University of Massachusetts Medical School Worcester, MA United States; ^2^ Department of Allied Health Sciences University of Connecticut Storrs, CT United States; ^3^ Department of Quantitative Health Sciences University of Massachusetts Medical School Worcester, MA United States; ^4^ Department of Obstetrics and Gynecology University of Massachusetts Medical School Worcester, MA United States; ^5^ Department of Emergency Medicine University of Massachusetts Medical School Worcester, MA United States; ^6^ Division of Health Informatics and Implementation Science Department of Quantitative Health Sciences University of Massachusetts Medical School Worcester, MA United States; ^7^ Department of Kinesiology & Community Health University of Illinois at Urbana-Champaign Urbana, IL United States; ^8^ Division of Consultation/Liaison Psychiatry and Psychology Department of Psychiatry Virginia Commonwealth University Richmond, VA United States; ^9^ Department of Psychology & Neuroscience Duke University Durham, NC United States; ^10^ Duke Digital Health Science Center Duke University Durham, NC United States

**Keywords:** mHealth, mobile health, mobile applications, telemedicine/methods, treatment efficacy, behavioral medicine, chronic disease

## Abstract

Commercial mobile apps for health behavior change are flourishing in the marketplace, but little evidence exists to support their use. This paper summarizes methods for evaluating the content, usability, and efficacy of commercially available health apps. Content analyses can be used to compare app features with clinical guidelines, evidence-based protocols, and behavior change techniques. Usability testing can establish how well an app functions and serves its intended purpose for a target population. Observational studies can explore the association between use and clinical and behavioral outcomes. Finally, efficacy testing can establish whether a commercial app impacts an outcome of interest via a variety of study designs, including randomized trials, multiphase optimization studies, and N-of-1 studies. Evidence in all these forms would increase adoption of commercial apps in clinical practice, inform the development of the next generation of apps, and ultimately increase the impact of commercial apps.

## Introduction

Mobile health (mHealth), or the use of mobile technology to improve health, is a rapidly expanding field [1]. As of 2015, more than 165,000 mHealth apps were available on the Apple iTunes and Android app stores, and 34% of mobile phone owners had at least one health app on their mobile device [2-4]. Although health apps have drawn great public interest and use, little is known about the usability and efficacy of the majority of commercially available apps [5,6].

Much mHealth research focuses on the development and testing of new apps in academic settings [7]. However, the pace of traditional academic research is slow and less nimble relative to commercial app development, and this may result in huge lags in dissemination into commercial markets or settings where the general public has access to them [8], assuming the researcher takes steps to disseminate into commercial markets at all. Producing an app for public use requires content, programming, design expertise, the ability to continually host and update the app, and the resources to provide both customer service and technical support [8-10]. Apps generally take 7 to 12 months to fully develop and launch and cost on average US $270,000 [10]. This does not include the added expense to maintain the app postdevelopment or the costs to publish the app to multiple platforms (eg, Apple and Android). Because many researchers will not have access to these resources, leveraging existing commercial apps in research may be an efficient and cost-effective alternative. The greater the scientific workforce dedicated to gathering evidence for health apps, the more quickly this field can evolve into one that is well grounded in evidence.

Health care providers also have great interest in determining the evidentiary basis of commercial apps. In fact, the American Psychiatric Association [11] and others [12] have developed guidelines for clinicians in selecting commercial apps to recommend to patients. A bedrock of these guidelines is that clinicians examine the evidence to make these decisions. With little evidence available for commercial apps, clinicians risk recommending a tool that does not work or worse one that causes harm. Although methods for systematically developing and establishing the effectiveness of apps in academic research laboratories have been described [13], little guidance is available on ways to develop an evidence base for commercial apps.

A recent systematic review provides a helpful starting point to describe methods that have been used in studies evaluating the quality of commercial health apps [14]. They report that among studies analyzing the quality of downloaded app content, methods used included rating apps relative to predefined criteria, rating apps relative to evidence-based criteria, and usability testing of functions [14]. Other studies analyzed content descriptions of apps using methods such as adapted website assessment tools, user ratings and reviews, and degree of involvement of experts in app development [14]. This review not only provides a useful overview of methods used in published studies but also points to the need for further work in developing and describing methods including those that have not yet been applied in research on commercial apps. We build on this work by detailing a wide variety of methods and study designs that can be used to evaluate commercial health apps.

The purpose of this paper is to present the full scope of methods for generating evidence for commercial health apps. Methods for evaluating commercial health apps reviewed include content analysis, usability testing, observational studies, and efficacy testing. Illustrative examples are used when possible to demonstrate the application of methods described; examples were identified using the results of PubMed searches with related terms (eg, mobile apps, content analysis, usability testing, observational study, and randomized controlled trial [RCT]). This review will also shed light on decisions regarding which methods match specific research question and the degree of time and resources involved in the various study designs. The identification of high-quality commercial apps is essential for research, clinical practice, and to inform the development of the next generation of commercial apps.

## Content Analysis

Content analysis is a research methodology that involves coding and interpreting qualitative, usually text-based material [15]. Commercial apps include multiple features, health information, and advice, all of which can be subject to content analysis. The first step in conducting a content analysis is to access the app content for review. In previous studies, the content that was analyzed came from either directly downloading the app and exploring its features or from the information provided in the app store (eg, app description and list of features) [14]. Although content analysis can simply involve describing the content included, another approach is to select a comparator against which the app content would be assessed. Three common comparators used in the scientific literature include clinical guidelines, evidence-based protocols, and behavior change techniques (see Table 1) [16-18]. Other possible comparators might include theoretical constructs or even other well-validated apps.

### Accessing Content

Content analyses of descriptions in the app store [19] or of content in the downloaded app [14] address different questions. Evaluating the app descriptions gives insight into the content that influences a user’s decision to download an app. A drawback is that app descriptions are not necessarily exhaustive sources of app content and may not exhaustively describe all features or content included in the app [19]. Coding the content of the downloaded app, on the other hand, will give insight into the actual content of the app. The drawback of this approach is that it may require some expense as many apps must be purchased. It also necessitates greater time investment as some apps require a period of use to experience all features. Content may also vary user by user as apps begin to employ artificial intelligence to personalize the content. Therefore, time, resources, and the research question must be considered when selecting an approach to accessing content for evaluation. Researchers should clearly articulate the limitations to the approach selected.

**Table 1:** Examples of evaluations of commercial mobile health apps.

Method and types of evaluation		Example studies		
		Health topic	Study aim	Findings
**Content analysis**					
	Clinical guidelines	Diabetes self-management [19]	Apps (N=227) evaluated for use of 7 self-management behavioral practices recommended by the American Association of Diabetes Educators	No apps promoted all 7 practices; 22.9% (52/227) included at least four of the practices, and 14.5% (33/227) did not include any practices
		Smoking cessation [20]	Apps (N=225) evaluated for use of the 5As clinical practice guidelines	51.1% of apps (115/225) implemented “ask,” 47.1% (106/225) “advise,” 8.0% (18/225) “assess,” 96.0% (216/225) “assist,” and 11.1% (25/225) “arrange follow-up”
		Pediatric obesity prevention and treatment [21]	Apps (N=57) examined for inclusion of 8 strategies and 7 behavioral targets recommended by the Expert Committee for Pediatric Obesity Prevention	61% (35/57) apps did not incorporate any evidence-based behavioral strategies; of the remaining 39% (22/57) apps, the mean number of strategies used was 3.6 (standard deviation [SD^b^] 2.7) out of the possible 15
	Evidence-based treatment strategies	Weight loss [22]	Apps (N=30) evaluated for inclusion of 20 evidence-based weight loss strategies used in the Diabetes Prevention Program	Apps included 19% (3.8/20) of the strategies
		Depression [23]	Apps (N=117) evaluated for incorporated cognitive behavioral therapy and behavioral activation treatment strategies	10.3% (12/117) of apps were coded as delivering any elements of cognitive behavioral therapy or behavioral activation
	Behavior change techniques	Physical activity [24]	Apps (N=64) reviewed for use of behavior change techniques	On average, apps included 22% (5/23) of the behavior change techniques (range 2-8)
		Physical activity [25]	Descriptions (N=167) for top-ranked apps evaluated for use of behavior change techniques	On average, App descriptions included 16% (4.2/26) of the behavior change techniques (range 1-13)
**Usability testing**					
	Laboratory studies	Multiple health outcomes (depression, diabetes, caregiving) [26]	Usability of apps (N=11) evaluated among diverse participants (N=26) through completion of a series of app-related tasks	42.7% (79/185) of tasks completed without assistance; participants were interested in using technology, but lacked confidence navigating the apps and were frustrated by design features
		Diabetes self-management [27]	Usability of apps (N=42) evaluated by two experts based on ease of use, user interface design, customizability, data entry and retrieval, integration of data into charts/graphs, data sharing	10% (4/42) of apps had a composite usability score above 20 (scale 1-30)
		Pain management [28]	Usability of apps (N=2) evaluated by patients with chronic pain (N=41) through recall of two pain memories; assessed for ease of use and time to enter pain data	Entry for the app Pain Scale was 89% faster than entry for the app Manage My Pain; Manage My Pain incorporated more attractive fonts and colors
	Field testing	Heart disease [29]	Usability of an app, Heartkeeper, evaluated through user feedback (N=26) on a survey that solicited feedback from existing users of the app in the field based on ease of use, performance, appearance, and perceived app security	Responses indicated that users were satisfied with the app
	User ratings	General patient-centered health [30]	User ratings for apps (N=234) evaluated for presence of 12 features; analyzed whether these features explained variation in user ratings of the app	Plans, ability to export user’s app data, general usability, and app cost associated with higher user ratings; presence of a tracking feature associated with low user ratings
				
				
				
				
**Observational studies**					
	N/A^a^	Mental health [31]	Evaluated data from users (N=152,747) of the stress reduction app Happify to explore whether greater usage predicted higher well-being	Greater app use predicted more positive emotion among app users
		Weight loss [32]	Examined cross-sectional associations between weight loss and components of weight loss app Lose It! use among app users (N=972,687)	People who used the app most often were more likely to achieve weight loss success of losing 5% of their starting weight (73% success) than those users who only used the app occasionally (5% success)
		Physical activity [33-35]	Three studies examined the associations between use of Pokémon Go and physical activity (two through survey and one through ongoing use of a physical activity device); an outcome external to the app	Use of the app was associated with short-term increases in physical activity
**Efficacy testing**				
	Randomized controlled trials	Weight loss [36]	Tested the effect of a weight loss app versus two traditional diet counseling methods (pen and paper and memo function on phone) on self-monitoring and weight loss among adults during an 8-week trial (N=57)	No between-group difference for weight loss; app condition participants kept more consistent diet records than pen and paper participants but not more than phone memo participants
		Weight loss [37]	Tested the effects of using MyFitnessPal weight loss app plus usual care versus usual care alone, for effects on weight loss and blood pressure over 6 months with N=212 primary care patients	No between-group differences found for weight loss or reduction in blood pressure differed between groups; app users set a calorie goal more often than the usual care group
		Smoking cessation [38]	Compared the efficacy of two smoking cessation apps over 8 weeks: a commercial app (QuitGuide) versus a researcher-developed app that incorporated Acceptance and Commitment Therapy	Researcher-created app was more effective than QuitGuide for quit rates (13% vs 8%) and participants engaged with it more than QuitGuide (opened app 37.2 times vs 15.2 times)

^a^N/A: not applicable.

^b^SD: standard deviation.

### Selecting a Comparator

#### Clinical Guidelines

Some content analysis studies have compared app content with clinical guidelines put forth by professional organizations (eg, Expert Committee for Pediatric Obesity Prevention) [39,19-21]. This approach can identify apps that are most comprehensive in their incorporation of clinical guidelines and identify gaps in the content of other apps. It can also lend credibility to commercial apps that score highly among researchers, clinicians, and patients [19]. Studies comparing the content of commercial health apps with clinical guidelines have found that guidelines are sparsely used (see Table 1) [19-21]. For example, 227 diabetes self-management apps were evaluated against seven self-management behavioral practices recommended by the American Association of Diabetes Educators [40]. Results revealed that no apps promoted all seven, 22.9% (52/227) included at least four, and 14.5% (33/227) of apps did not include any of the behavioral practices [19]. However, as the researchers suggest, it is unlikely that all users will need or want every aspect included in clinical guidelines; for example, some patients may want to track their medications, whereas other patients may not be on medication [19]. Although commercial apps may not incorporate all components of clinical guidelines, they can still be useful tools to deliver some key components of the guidelines. Understanding which components of the guidelines are included can help users and providers select the app that best matches their needs. One challenge for app developers is that clinical guidelines change as the science evolves, and some changes are heavily debated among scientists and practitioners (eg, American Heart Association dietary fats recommendations) [41], which can be confusing for developers and users. Staying abreast of changing guidelines would be necessary to insure that information provided is current.

#### Evidence-Based Protocols

Another comparator for commercial app content analysis is an evidence-based protocol. An evidence-based protocol is a structured collection of behavioral strategies that when implemented together and as recommended have produced significant effects on behavior or a health condition in randomized trials (eg, Diabetes Prevention Program Lifestyle Intervention) [42]. A comparison of apps with evidence-based protocols can provide useful information about the strategies being deployed. To date, studies comparing the content of commercial health apps with evidence-based protocols have consistently found low rates of strategies included (See Table 1) [22,23]. For example, one study evaluated 30 weight loss mobile apps for inclusion of the 20 evidence-based weight loss strategies used in the Diabetes Prevention Program lifestyle intervention protocol (eg, weight loss goal, portion control, problem solving, and stress reduction) [22]. Overall, the apps included only 19% (3.8/20) of the strategies, but nearly all apps (93%) included setting a weight loss goal [22]. These findings suggest that although commercial apps do not generally appear to be providing a comprehensive set of behavioral strategies, they may assist the user with specific behavioral strategies.

#### Behavior Change Techniques

Another approach to analyze the content of apps has been to identify and classify the behavior change techniques used in the apps. A taxonomy of behavior change techniques was developed through a systematic process where health behavior theories and meta-analyses of interventions were reviewed to generate a list of discrete evidence-based techniques (eg, prompt barrier identification, model or demonstrate the behavior, and plan social support) [17]. The goal of the taxonomy is to provide a list of behavior change techniques in their smallest reducible size and to improve the specification, replication, and implementation of behavioral interventions [16-18]. Numerous validation studies have shown that researchers can use the taxonomy to reliably classify behavior change techniques [17,43]. Furthermore, research has shown that certain behavior change techniques are associated with more favorable outcomes [17,44,45]; therefore, evaluating apps for inclusion of these behavior change techniques could aid in identifying appropriate apps for specific behavior change goals. Two studies have evaluated the content of commercial physical activity apps to describe their utilization of behavior change techniques [24,25]. One study found that, on average, physical activity apps incorporated 5 of the 23 behavior change techniques (22% of total) [24]; another one found that app descriptions mentioned, on average, 4.2 of the 26 behavior change techniques (16% of total) [25]. As more behavior change techniques are implemented in commercial apps, behavioral providers may be able to give tailored recommendations of apps to match patients’ specific behavioral challenges.

### Challenges to Content Analysis

Content analyzing commercial apps can be challenging for four main reasons. The first challenge is the variability in the way apps implement clinical guidelines, evidence-based strategies, and behavior change techniques. For example, an app might implement goal setting by allowing a user to set a behavioral goal. Goal setting implemented during behavioral counseling would not only involve the individual selecting a goal but would also provide assistance with selecting realistic and measurable goals and guidance on adjusting the goal over time based on the individual’s performance. In this case, the app developers would have to make a judgment call as to whether goal setting in the app reached the fidelity threshold for goal setting as originally intended. When evaluating the content of apps, researchers are encouraged to specifically describe the threshold for each behavioral strategy. Continuous rating scales could also be used instead of simple yes or no” indicators of the presence of a strategy to more fully capture the extent to which the strategy was implemented.

A second challenge to content analysis is that methods presented here rely on subjective ratings of app content and app features. A recent study demonstrated the difficulty of conducting consistent assessments of app content between reviewers, as evidenced by low interrater reliability scores [46]. Researchers are cautioned to use tools that involve little reviewer discretion (ie, assessed on a factual basis) to reliably evaluate app content and features across individuals [46].

A third challenge to content analysis is that apps are frequently updated which may result in continuously changing features, loss of features, and new features. The app version number and download and review dates should be disclosed in content analysis reports. Given how often companies release app updates, content analysis reviews can quickly become obsolete and may need to be performed quickly and frequently.

A final challenge to content analysis is that some apps release features only after a period of use or with an additional cost [22]. The period of use may be based on time spent or accomplishment of specific goals. These features might be missed if coding is only done in a single use episode or without purchasing the extra features. Therefore, proper recording of the duration of use and presence of additional paid features in apps is recommended.

## Usability Testing

Usability or user testing refers to how well an app functions and whether or not it serves its intended purpose. Typically, usability is measured across dimensions such as user ratings of app flexibility, operability, understandability, learnability, efficiency, satisfaction, attractiveness, consistency, and error rates [47-51]. Usability testing specific to a target population can be particularly helpful for researchers or clinicians whose work focuses on those populations [47]. The International Organization for Standardization (ISO) is a leader in developing industry standards and evidence-based guidelines for the development of a range of services and products, including technologies [52]. Two recent International Standards (ISO 9241 and ISO 25062) provide guidelines for conducting and reporting on usability testing of mobile apps [53]. These standards frame usability testing and results in terms of the feedback from users, as opposed to past standards that defined usability based on the software product itself [53]. Developers may approach the process of usability evaluation through methods such as experts-based evaluation (ie, experts describe the problems that users might encounter), observation (ie, watching users interact with the app), surveys (ie, to collect user feedback), and experimental evaluation (ie, evaluation of a product through interaction with app by experts or users to collect feedback on usability issues) [47,53]. Evaluation of commercial app usability can include laboratory testing, field-based evaluations, and reviewing ratings and narrative user reviews from app marketplaces (Table 1).

### Laboratory-Based Testing

Usability testing can be conducted in a laboratory where users are asked to carry out specific tasks with an app in a controlled setting with extensive observation [51]. Laboratory-based testing can be helpful, especially when usability needs to be assessed in a specific population who may have different characteristics than the users targeted by the company (see Table 1 for examples) [26-28]. Usability metrics, such as comprehensibility and ease of use, can be collected over a short period of time with a small number of people. In a single visit, laboratory-based usability testing can provide rich data by allowing user behavior to be audio- or video-recorded. Investigating the way that members of the target population click through and understand various screens and features may uncover usability issues [47]. For example, a researcher might be interested in identifying a commercial exercise app that has high usability in older cancer survivors. Results from laboratory-based testing can be used to inform the instructions and training given to the target population or additional technology needed to support use of the app. For example, investigators might be able to design workarounds for app deficiencies (eg, use mobile phone settings for color changes and font size to make app more readable) to boost their usability in future research. One limitation of usability testing is that it may not represent how users will interact with the app in the real world [28,51]; therefore, more extensive field testing may be necessary.

### Field Testing

Field testing or mobile in the wild testing allows observation of how people use the app in their real lives [51] to better understand real-world usage of the app [54,55]. Testing apps in the field can test usability of an app for a specific target population or help determine which of the several apps is best for a target population. Few studies have used field-based methods to evaluate the usability of commercial health apps (Table 1) [29]. One study evaluated the app Heartkeeper by incorporating a button into the app where users could click and complete a quality of experience survey to rate content quality, security, ease of use, availability, performance, appearance, and learning of the app [29]. Responses indicated that users were satisfied with the app [29]. Another method to collect field usability data is through app tracking software. Software can be installed on mobile phones to monitor the number of active app users, how long users spend in the app, what they click on, and so on. Researchers should consider utilizing these programs and reporting on app use data to supplement other field testing results. Despite the rich data field tests can provide, capturing app use in a dynamic environment makes direct observation difficult [21]. Furthermore, findings may only be relevant to the sample of users selected and samples tend to be small [26]. Additional evidence for app usability in a variety of populations is critical to provide further insight into which apps might be best suited for whom.

### User Feedback: Ratings and Reviews

User feedback on the app marketplace is a source of usability data that reflects the experiences of people who presumably downloaded and used the app. These data can demonstrate app popularity via total number of ratings, as well as quality via average rating (typically as a number of stars out of 5) and narrative reviews. Although mean rating provides an overall estimate of quality or desirability, the distribution of ratings may be important to understanding the mean rating. For example, an average rating of 3 stars could either suggest that most ratings hovered around 3 stars, or could be reflective of highly polarized ratings (ie, mostly 1-star and 5-star ratings). Low ratings may indicate a specific issue with the app or contradictory opinions of the app overall. Ratings may change over time because of updates (eg, bug patches and function improvements) and users changing their past ratings over the course of app use (as allowed by some app stores). However, recent research suggests that caution should be taken when interpreting these ratings as they are correlated with unexpected factors such as time to last update, app vocabulary, and the app description [56]. Narrative reviews can provide qualitative data about the positive and negative aspects of usability, user interface, and match between intended use and functionality. Reviews may also include users’ perceptions of efficacy (eg, “this app is great!! I lost 10lbs using it!!”). Because not all users provide reviews, reviews may oversample highly positive and negative experiences rather than the “average” experience. Content analysis [57], sentiment classification, and natural language processing may be useful for examining user-narrative reviews. One limitation is that app creators can write reviews themselves or otherwise incentivize users to give favorable ratings, affecting interpretability of these data [14].

## Observational Studies

Observational studies can be used to assess app use, satisfaction, and the predictive value of app use on behavioral and clinical outcomes. Observational studies can be conducted via large databases of users or case series of a small number of users to assess outcomes tracked by the app (Table 1) [31-35]. Although observational studies cannot establish causality (ie, efficacy of the app on an outcome), they can be used to explore associations between app use and outcomes. For example, an observational study of users of popular weight loss apps might examine whether length of use is associated with greater weight loss. Observational studies can also provide information about duration of use in real-world settings for specific types of users [58]. For example, ecological momentary assessment can be utilized to gather data numerous times throughout a day [59] to provide information about use patterns across people or intraindividual use patterns. A limitation of observational studies is the potential for selection bias, especially when examining prolonged use of the app and the inability to draw causal conclusions about observed behavior changes. Additionally, app users are not likely representative of patient populations (eg, MyFitnessPal users likely have different characteristics than primary care patients with obesity). Furthermore, information regarding the characteristics of users may be limited, making it difficult to ever know whom the data represent. For this reason, it would be important to clearly describe the limitations of the data in manuscripts and other public reports. Given the massive amount of data companies have on the use of their apps, observational studies present an enormous opportunity for academic-industry collaboration. Academics could partner with companies who are interested in having their outcome (eg, weight loss and physical activity) and process data (eg, self-monitoring patterns) analyzed. Alternatively, companies are increasingly hiring behavioral and data scientists to explore their data, providing a novel industry career path for academics looking to use their skills to inform commercial products.

## Efficacy Testing

Efficacy testing is a critical step in establishing whether use of a commercial app results in meaningful change in behavior and clinical outcomes. The gold standard approach to efficacy testing is the RCT [60]. However, given the time and expense required to perform RCTs, alternative study designs like N-of-1 and case series can be considered as initial steps to justify the progression to RCT.

### Randomized Controlled Trials

Evidence from RCTs (Table 1) is considered the gold standard in the context of clinical guidelines [61], which is ultimately the gateway to becoming a part of standard practice. A major decision point in RCTs is the appropriate control or comparison group with each option addressing a unique question. Usual care control groups address whether a commercial app improves upon usual care [37]. On the other hand, one might be interested in testing whether an app-delivered behavioral strategy improves upon the same behavioral strategy when delivered via a traditional modality (eg, dietary self-monitoring via app vs paper diaries) [36], in which case a noninferiority trial using the traditional condition as comparator is appropriate. If the research question is whether an app improves upon a standard practice, a comparison could be made between standard practice with and without the app [37]. Comparative effectiveness studies including both equivalence and noninferiority designs might compare two apps or an app with another treatment approach. For example, one RCT tested whether a new investigator-generated smoking cessation app utilizing a novel behavior change model was more effective than a commercially available app [38].

#### Challenges

RCTs are time and resource intensive, which means their use must be reserved for apps in which other previously discussed forms of evidence support the investment. Another challenge to RCTs with commercial apps is that frequent app updates make it difficult to ensure that all participants receive identical intervention. Treatment fidelity and receipt should be tracked so that such deviations can be documented and controlled for in analytic models. Finally, researchers have no control over the features in a commercial app, making it difficult to test whether the “success” of an app-delivered intervention is attributable to the total package of the app or because of specific app components.

### Alternative Study Designs

#### Optimization Strategies

To address research questions about the efficacy of individual app features, researchers may consider utilizing an optimization design, such as the one described in the multiphase optimization strategy (MOST) framework [62,63]. The MOST framework is an iterative research design that allows investigators to select and evaluate individual components, rather than the treatment as a whole, to optimize the effect of individual components on behavior change. Specific study designs within this framework include factorial designs and sequential multiple assignment randomized trials [62]. Furthermore, parallels have been drawn between the use of optimization designs, such as MOST, for behavioral trials and the process used for software development, which is described as an “agile science” process for behavioral research [64]. The agile science process calls for researchers to target and test specific components of new products (eg, apps) for rapid testing of and adaptation to the smallest meaningful unit possible, allowing for more efficient iteration and dissemination [64]. The MOST framework has yet to be applied to testing the efficacy of commercial apps, and one challenge is in randomizing participants to only using parts of an app when they have access to the entire app. This work might ideally be performed during the design phase of the app in the context of an academic-industry partnership. Studies could leverage a MOST design to test different combinations of commercial apps that each provide a unique behavioral strategy; however, efforts would need to be taken to prevent contamination as commercial apps are publicly available.

#### N-of-1 Studies

A fairly quick way to build efficacy data for a commercial health app is via N-of-1 designs. This methodology, also known as “single-case,” involves the repeated measurement of an individual over time and is a practical method for understanding within-person behavior change after presenting an intervention (ie, AB design) or after presenting the intervention and then removing it (ie, ABA design). Similar to the process recommended by researchers to rapidly iterate mobile app development in the laboratory [8], N-of-1 trials could be used to test the preliminary efficacy of established commercial apps using methods analogous to personalized medicine (ie, iterative crossover designs) [65]. For example, those interested in testing whether exposure to theory-based content of a healthy eating app influences the dietary choices of individual participants might use a series of ABA N-of-1 designs to describe intraindividual variation in behavior before and after exposure to that feature. Furthermore, ongoing work in dynamic statistical modeling provides guidance for analyzing the data from N-of-1 trials [66], including techniques to increase the generalizability of estimates [67]. Although no published studies have used N-of-1 designs for testing commercial apps, a recent systematic review examined the evidence for using N-of-1 studies for other health behavior interventions, describing the current state of evidence supporting N-of-1 studies, and methodological considerations for designing and executing N-of-1 studies [68]. The review also offers insights about the potential for technology to help collect large amounts of individual data from participants both unobtrusively and longitudinally [68]. N-of-1 designs do have important limitations, including lack of generalizability, limited consensus on appropriate analytic techniques, and failure to address long-term maintenance of behavior change. Additionally, use of N-of-1 designs for testing mobile apps include the potential to overestimate effects because of the so called “digital placebo” effect, which is the ability of expectations of the benefit of using a digital tool such as an app to lead to clinical improvement [69]. The digital placebo effect could partially explain consumers’ reports of benefits from apps that are largely devoid of evidence-based strategies and unlikely to provide substantive benefit [69]. Researchers employing an N-of-1 design are cautioned to account for these limitations in their study designs.

## Discussion

In this paper, we described a host of methods that can be used to systematically evaluate commercial apps as a way to stimulate a science of commercial health apps. Greater evidence for commercial apps could increase their adoption in clinical practice and impact on behavioral and clinical outcomes. Commercial apps are typically developed with a high level of expertise in design and function and many are well marketed and have enormous user bases. Scientists who do not have the resources to develop their own apps can instead employ less resource–intensive research on commercial health apps. Industry professionals and investors would benefit from data on the content, usability, and efficacy of the commercial apps to inform their decisions on future products and investments.

### Future Research

Additional areas of exploration in researching commercial apps may include evaluation of the technical functions of the app, developer transparency, and policies regarding user data privacy and security (eg, transparency about how developer will use app data) [70]. In terms of technical performance of the app, research could evaluate features such as validation of information inputs (eg, app verifies that the information a user inputs is plausible or flags the entry and asks for a correction) and information security precautions (eg, whether user’s medical data are susceptible to interception) [70]. In terms of developer transparency, researchers could use app metadata to extract manufacturer information, contact information, and product information. For example, do manufacturers include professional expertise in the target health area, such as endocrinologist for a diabetes self-management app? These data would also allow researchers to evaluate relationships between app quality, user ratings, and developer transparency [56,71]. Another important dimension of transparency is extent of user information required to run the app and whether permissions requested are necessary. A recent review investigated the declarations of manifest files and app source code to determine whether the permissions requested were related to the information needed to run the app [72]. Results suggested that requested permissions often surpassed what the app needed, which means these apps could pose an unnecessary threat to user privacy and safety [72]. In terms of evaluation of the privacy and security of commercial apps, researchers can track whether users retain the rights to their own data, whether data are adequately protected during transmission and storage, and developer transparency (eg, published contact information if users have questions) [73]. A growing interdisciplinary dialogue is emerging about the ethical considerations of using health technologies, including proper precautions that should be taken to ensure user privacy and safety [73,74].

### Limitations

This review has some limitations. First, we did not conduct a systematic review of app evaluation studies, but rather present a focused summary of methodologies commonly used in studies testing traditional interventions with details on how they can be applied to commercial apps, with illustrative examples where possible. In general, another limitation of this review is that commercial products may be updated, completely changed, or discontinued while a research study is in progress, making findings obsolete before they are even published. Apps that were developed by established companies, have been in the marketplace for a while without major changes, and have large and devoted user bases may be less likely to change drastically over the course of a research study. Research on a commercial app that contains features that are common to many other commercial apps will have relevance to those other apps even if the target app no longer exists. However, the rapid pace of technology means researchers should avoid delays in data analysis and publication for this work. Historically, traditional interventions have evolved relatively slowly, which allowed lags in the research process. Such lags cannot be afforded for this work. To speed the process, researchers should be sure to establish a firm project timeline, select collaborators who are willing to commit to the project timeline, and target journals with fast review turnaround times and brief report article types.

### Conclusion

Research on commercial mHealth apps can take many forms depending on the research question as well as the time and resources required to complete it. No single methodology is best as each provides a different type of evidence and involves a unique set of advantages and limitations. Research on commercial mobile apps complements research exploring the development and testing of novel apps in academic laboratories. Both have a place in the literature and together will propel the mHealth space forward and strengthen the degree to which its foundation is empirical evidence.

## Abbreviations

ISO: International Organization for Standardization
mHealth: mobile health
MOST: multiphase optimization strategy
RCT: randomized controlled trial
SD: standard deviation
N/A: not applicable
